# Supplementary material for: Personalized models of disorders of consciousness reveal complementary roles of connectivity and local parameters in diagnosis and prognosis
Source: PLoS One. 2025 Sep 2;20(9):e0328219. doi: 10.1371/journal.pone.0328219 (PMC12404413; doi:10.1371/journal.pone.0328219)
Supplement: Attachment — Submitted filename: PONE-D-24-54958_reviewer_GR.pdf [file pone.0328219.s002.pdf]

Review PONE-D-24-54958

The manuscript presents a framework for modeling disorders of consciousness (DoC) at the individual patient level by combining dimensionality reduction techniques and biologically inspired whole-brain modeling. The main finding—that connectivity matrices and local parameters carry complementary information regarding the patient's diagnosis and prognosis—is highly relevant.

The strengths of this study are manifold. The latent space framework, employed in recent publications from the same team, simplifies data complexity while retaining essential diagnostic features, demonstrating robustness across different datasets, including the two DoC datasets analyzed in the present manuscript.

Connectivity-based brain models are then simulated to reproduce the latent dynamics in silico. The authors employ a recently introduced astrocyte-inspired model (AHP) alongside the more established Hopf model. Integrating biologically meaningful parameters, such as those derived from the AHP model, is another major contribution, as it offers a pathway to understanding underlying brain mechanisms disrupted in DoC, as the authors underscore in the discussion. The authors have made the pipeline available, which helps transparency.

The potential applications of model-based biomarkers (e.g., connectivity and local node parameters), both in clinical decision-making and personalized treatment, highlight the broad interest of this work.

Despite its strengths, the manuscript needs significant revisions to enhance clarity and methodological robustness.

- One major issue pertains to the connectivity analysis and its application across datasets. It is unclear whether the authors applied the analysis to both the Paris and Liege datasets or only to one of them. If (as I understand) both datasets are used for the results in Figure 3, mixing CNT, MCS, and UWS groups from two different datasets could result in spurious classification, as the datasets are acquired at different resolutions using different pipelines (despite the latent space method, which might partly suppress these differences). Confirming the effectiveness of the classification separately on the two datasets would strengthen the validity of the results.

- Another area that needs further clarification is the interpretation of connectivity results. The manuscript identifies positive and negative connections within the generative effective connectivity (GEC) matrices but stops short of exploring their interpretation. A discussion on the meaning of positive inter-clique links and negative intra-clique links would enrich the study. For instance, mapping the latent modes back to standard resting state networks (as in Sanz-Perl et al Network Neuroscience 2025) could provide neuroscientific interpretability, helping to connect the findings (about the modular interaction of two cliques) with existing functional network frameworks.

- The clustering of patients into groups with different outcomes is intriguing but requires a more detailed quantification. For example, the interpretation of Fig.4C reports that "*clusters 2 and 3 contain mainly patients with negative outcomes, cluster 1 contains a majority of emerged patients, and clusters 0 and 4 contain more mixed outcomes*". However, there are no numbers displayed to assess if the percentages support the analysis by visual inspection.

- The manuscript does not provide a rationale for the choice of fixing the global coupling (G=1). Since G is generally regarded as a crucial parameter that influences global network dynamics, wouldn't it be beneficial to include it in the iterative fitting process alongside local parameters? It would be helpful if the authors provided a justification for why G is held constant.

- The Methods section would benefit from greater transparency and detail. Key metrics, such as the structural similarity index (SSIM), are referenced but not explicitly defined. Including a brief description of the SSIM formula would make the study self-contained. Similarly, the procedures for fitting model parameters and the rationale behind the specific GEC formula are not described with sufficient clarity. While it is reasonable to reference prior work for context, the paper would be easier to follow if these core methods were explicitly presented.

- Beyond methodological issues, the manuscript's writing could be improved to enhance readability. The use of inconsistent tenses, punctuation errors, and occasional typos (e.g., "*… simulations is* N_{max} *is reached*", or "*. Where the shifted matrices are obtained..*", or "*using the* $edge_{b}$*oundary function*") detract from the clarity of the scientific content. I recommend using tools like Grammarly to identify and correct these issues. Additionally, some sentences are overly verbose or unclear, such as "*Moreover, analysis of the clusters' separability also revealed which parameter, or group of parameters, were leading to belong to which cluster*" which could be rephrased more concisely. A thorough revision for grammar, style (e.g., reducing the excessive use of the Saxon genitive 's), and logical flow would make the paper more accessible to the reader and better communicate its scientific value.

- A revised title might help improve the paper's visibility. The current title, "*Modeling disorders of consciousness at the patient level reveals the network's influence on the diagnosis vs the local node parameters role in prognosis*", feels a bit lengthy. Additionally, using "vs" is not ideal. You might consider something like: "*Personalized models of Disorders of Consciousness reveal complementary roles of connectivity and local parameters in diagnosis and prognosis*".

- The figures, while generally informative, also require some adjustments. Missing labels, such as for the clusters' IDs, or the absent color bar in functional connectivity figures, should be added to ensure clarity. In Figure 4A, the yellow cluster is nearly invisible, making it difficult to interpret the results. Adjusting the color palette or improving the visual contrast would address this issue. Figure S2 title should be "*GEC-based biomarkers for the Hopf model:*" and panel C should be adjusted in the caption "*bf C.*".

- Notice that PONE guidelines specify that "If there are restrictions on publicly sharing data […] those must be specified."

In conclusion, this study presents a promising framework for analyzing DoC patients, combining innovative methodologies with biologically grounded insights. However, substantial revisions are required to address the issues outlined above. Expanding the connectivity analysis to both datasets or justifying their combined analysis would enhance the manuscript's robustness, while clearer interpretations and improved methodology transparency will help realize the full potential of this promising framework. I hope that these comments will assist in improving the manuscript.

Sincerely,

Giovanni Rabuffo
