# Supplementary material for: Personalized models of disorders of consciousness reveal complementary roles of connectivity and local parameters in diagnosis and prognosis
Source: PLoS One. 2025 Sep 2;20(9):e0328219. doi: 10.1371/journal.pone.0328219 (PMC12404413; doi:10.1371/journal.pone.0328219)
Supplement: Attachment — Submitted filename: Review PONE-D-24-54958-09052025.pdf [file pone.0328219.s003.pdf]

<u>Review PONE-D-24-54958</u>

The manuscript presents a framework for modeling disorders of consciousness (DoC) at the individual patient level by combining dimensionality reduction techniques and biologically inspired whole-brain modeling. The main finding—that connectivity matrices and local parameters carry complementary information regarding the patient's diagnosis and prognosis—is highly relevant. The strengths of this study are manifold. The latent space framework, employed in recent publications from the same team, simplifies data complexity while retaining essential diagnostic features, demonstrating robustness across different datasets, including the two DoC datasets analyzed in the present manuscript.

Connectivity-based brain models are then simulated to reproduce the latent dynamics in silico. The authors employ a recently introduced astrocyte-inspired model (AHP) alongside the more established Hopf model. Integrating biologically meaningful parameters, such as those derived from the AHP model, is another major contribution, as it offers a pathway to understanding underlying brain mechanisms disrupted in DoC, as the authors underscore in the discussion. The authors have made the pipeline available, which helps transparency.

The potential applications of model-based biomarkers (e.g., connectivity and local node parameters), both in clinical decision-making and personalized treatment, highlight the broad interest of this work. Despite its strengths, the manuscript needs significant revisions to enhance clarity and methodological robustness.

• One major issue pertains to the connectivity analysis and its application across datasets. It is unclear whether the authors applied the analysis to both the Paris and Liege datasets or only to one of them. If (as I understand) both datasets are used for the results in Figure 3, mixing CNT, MCS, and UWS groups from two different datasets could result in spurious classification, as the datasets are acquired at different resolutions using different pipelines (despite the latent space method, which might partly suppress these differences). Confirming the effectiveness of the classification separately on the two datasets would strengthen the validity of the results.

<u>Answer:</u> We indeed used both datasets (Paris and Liege). Several reasons justify this choice:

- To adjust for the different time resolutions from the different recordings, we performed a time interpolation step to ensure that all time-series had a similar time resolution. This is done using the *interpolate_timestep* function provided in the codes, which calls the *interp1d* function from *scipy.interpolate* and we selected the same time-length for all datasets to have the same number of points for all subjects.
- The preprocessing methods used to extract the time-series from the fMRI data were performed by the same person using the same pipeline.
- The ground truth classification used in the manuscript comes from the clinical diagnosis performed by the expert clinicians in Paris and Liege based on the COMA RECOVERY SCALE-REVISED (CRS-R) method, which is a universal framework for clinical diagnosis developed for that purpose to ensure that the diagnosis of DoC patients is consistent between different centers. It does not depend on the fMRI data or the scanner used to record it.

- Any classification algorithm trained on only one dataset would more likely pick up some specific artifacts or features specific to the scan setup used, and resulting in overfitting for this specific dataset. Mixing different datasets allows us to avoid this pitfall and ensure generalizability of our method, regardless of the center and technical setup where the data is recorded.
- As suggested, the dimensionality reduction and the projection in the latent space does indeed help to ensure that specific artifacts that could be due to the data recording be suppressed or at least reduced enough not to interfere with our results.
- Moreover, splitting the datasets would greatly reduce the number of patients in each group, thus the statistical power and significance of any group-related analysis.

We have now added **paragraph 3.6.1 p.15** in the methods section to clarify this point:

''In all that follows we combined the datasets from Paris and Liege described above in sections 3.2 to 3.4.

To overcome the problem of different time resolutions between the different datasets, we performed a simple time-interpolation step on the subsets with larger TR to obtain time-series that all have the same TR = 2s. This was done using the interp1d *function from the* scipy.interpolate *package.*

The decision of combining the different datasets was taken for the sake of robustness. Indeed, any classification algorithm trained on only one dataset would likely pick up some artifacts or features specific to the scanner used. This could result in overfitting to this specific dataset. Mixing different datasets allows us to avoid this pitfall and ensure the generalizability of our methods, regardless of the center and technical setup where the data was recorded. Furthermore, the preprocessing was strictly the same for all recordings from both centers, thus ensuring no differentiation between the datasets at this stage. Moreover, combining the datasets greatly increases the number of patients in each group and thus the statistical power and significance of any group-related analysis. We recall that the group classification (between MCS and UWS) used in the manuscript comes from the clinical diagnosis performed by the expert clinicians in Paris and Liege based on the CRS-R method which is a universal framework for clinical diagnosis developed for that purpose to ensure that diagnosis of DoC patients is consistent between different centers. Finally, the dimensionality reduction also reduces possible differences due to the differences in data recording.''

• Another area that needs further clarification is the interpretation of connectivity results. The manuscript identifies positive and negative connections within the generative effective connectivity (GEC) matrices but stops short of exploring their interpretation. A discussion on the meaning of positive inter-clique links and negative intra-clique links would enrich the study. For instance, mapping the latent modes back to standard resting state networks (as in Sanz-Perl et al Network Neuroscience 2025) could provide neuroscientific interpretability, helping to connect the findings (about the modular interaction of two cliques) with existing functional network frameworks.

Answer: This is a good point. To address it, we performed a new analysis similar to what was done in *Sanz-Perl et al. Network Neuroscience (2025),* except that instead of generating surrogate signals with white noise on each latent dimension, we took the actual signals from the control subjects. Briefly, we encoded all the control subjects and then, for each one, we kept only the encoded signal on one of the latent dimensions (LD in [|0,14|]) and set the other ones to zero. Then, we decoded those signals with activity on only one of the latent dimensions, for all latent dimensions. After decoding, we observed that, as expected, all decoded signals per latent dimension had a similar

structure, regardless of the dataset (Paris or Liege). Thus, we averaged over all the control subjects (from both datasets to be consistent with the rest of the study).

Then, we computed the SSIM between the decoded FC matrices for each latent dimension (i.e., the pairwise Pearson correlation coefficient of the decoded signals) and the correlation matrices obtained for each of the 7 resting state networks (RSNs) from *Yeo et al. (2011)*, i.e., the matrices obtained by keeping the control subjects' data in the nodes corresponding to one of the RSNs and putting very small amplitude white noise on the other dimensions. The very small amplitude noise, instead of zeros, is there to avoid the division by zero in the computation of the correlation coefficient for the signals that would have a variance equal to zero otherwise.

Finally, we did the same study but for the two cliques observed in Figure 3 on the GEC matrices' structure (i.e., instead of keeping the encoded signal only one latent dimension at a time, we kept the signal on all the nodes of one clique and then of the other one).

This allowed us to uncover links between specific RSNs and specific dimensions in the latent space, in particular, Latent dimensions 2 and 13 are strongly associated to the somato-motor network, while Latent dimensions 1 and 9 correspond to the default mode network (DMN), latent dimension 5 is associated to the dorsal attention network, while ventral attention is more related to dimension 11.

On the other hand, the visual network is not clearly associated with only one or two latent dimensions, but is strongly associated with Clique 0. Altogether, these results indicate that we can associate a *combination* of latent dimensions with the different RSNs more than one in particular.

We decided to use only the control subjects here because the severe brain lesions from the patients would make these structural results very difficult to interpret in the case of the patients.

These results are now summarized in the **new Figure 4**, and fully described in the new **results paragraph 1.3.2 p.8-9**:

"To further understand the meaning of the two-clique structure observed in the GEC matrices (fig. 3B), we decoded the signals from the latent GEC cliques and each latent dimension to see how they correlate to the natural structures in the brain. In particular, we investigated the link between GEC cliques (resp. latent dimensions) and the 7 Yeo Resting State Networks (RSN) [Yeo et al. 2011], with a similar approach to the one used in [Sanz-Perl et al. 2024] (see Methods, section 3.10).

Briefly, we decoded signals with activity (from the control subjects) on only one of the two GEC cliques. We computed the resulting FC matrix (fig. 4A, left, and B) for the average over all control subjects. We then compared these two FC matrices with those corresponding to activity on each of the 7 RSNs (fig. 4A, right). For both cliques, we computed the SSIM between the clique FC and each RSN FC (fig. 4C). We found that the visual network (light gray) projected most strongly on Clique 0, followed by the Dorsal Attention network (blue). In contrast the default mode network (DMN, beige) and the Ventral Attention network (purple) were more strongly associated with Clique 1.

We then decided to investigate the connection between each latent dimension LD in [|0,14|] and the 7 RSNs. Using a similar approach, we compared the FC matrices from decoded signals with activity on only one of the LD at a time to the RSN FC matrices (fig. 4D). We found that the Somatomotor network (light blue), was most strongly associated with latent dimensions LD 13 and 2, while the DMN strongly projected onto LD 1 and 9. We also noted that LD 11 was mainly related to the Ventral attention network. Generally, most networks were associated with a combination of latent dimensions rather than one in particular.

This could mean that the lower within-clique density observed in UWS patients is related to lower or less consistent activity on specific RSNs. However, a more detailed analysis would be required to confirm such a hypothesis.

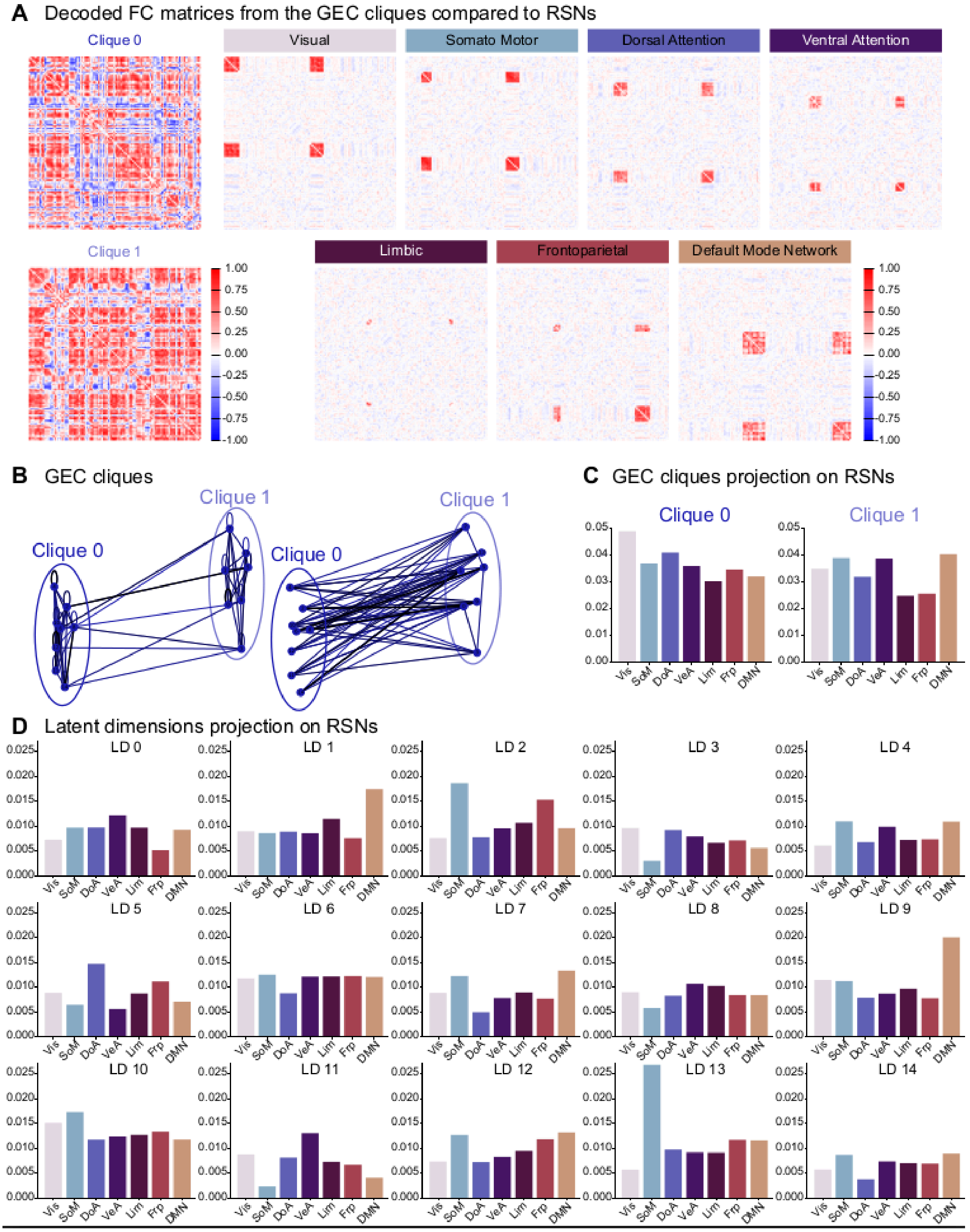

***<u>New figure 4:</u> GEC cliques and latent dimensions connection to the Resting State Networks**: **A.** FC matrices from the decoded signals for both GEC cliques (left), and FC matrices corresponding to the 7 RSNs in the natural space. **B.** GEC cliques from the average CNT GEC graphs. **C.** SSIM between the decoded FC matrices from each GEC clique compared to each RSN. **D.** Same as C but for the decoded signals from each latent dimension.*

as well as in the **<u>methods paragraph 3.10 p.20</u>**:

"To understand the connection between the latent dimensions and the natural brain structures, we created surrogate signals in the natural space corresponding to each latent dimension, as follows:

1. *We projected all the control subjects' data into the latent space, using the same trained AE as for the rest of the study.*
2. *From each encoded subject, we created d_{opt}=15 surrogate signals, by keeping the encoded data on one of the latent dimensions LD in [|0,14|] and setting the other ones to zero.*
3. *We decoded the surrogate signals (with the decoder part of the same trained AE) to project them back into the natural space. At this stage, we obtained 15 surrogate fMRI signals (with 100 time-series - the dimension of the natural space) per control subject.*
4. *We averaged the decoded fMRI signals over all control subjects. This was done to strengthen the results' robustness, after we verified that, as expected, all decoded signals per latent dimension had a very similar structure, regardless of the dataset (Paris or Liege).*

Then we compared the obtained signals for each latent dimension to the 7 resting state networks (RSNs) from [Yeo et al. 2011]. To do so, we computed a FC matrix for each RSN, in the natural space, by keeping the control subjects' data only in the nodes corresponding to one of the RSNs and putting very small amplitude white noise on the other dimensions (generated with 10^{-3} x numpy.random.rand*). The small noise on the remaining dimensions was added to avoid the division by zero in the computation of the correlation coefficient for signals that would otherwise have a variance equal to zero.*
Finally, we computed the SSIM between the decoded FC matrices for each latent dimension (LD in [|0,14|]) and the FC matrices obtained for each of the 7 RSNs.
We applied the same methodology for the two cliques of the GEC observed in Fig. 3B. This means that instead of keeping the encoded signal only one latent dimension at a time, we kept the signal on all the nodes of one clique and then of the other one. The rest of the analysis process was kept the same."

• The clustering of patients into groups with different outcomes is intriguing but requires a more detailed quantification. For example, the interpretation of Fig.4C reports that "clusters 2 and 3 contain mainly patients with negative outcomes, cluster 1 contains a majority of emerged patients, and clusters 0 and 4 contain more mixed outcomes". However, there are no numbers displayed to assess if the percentages support the analysis by visual inspection.

<u>Answer:</u> We have summarized all the quantifications in **<u>Table 1 p.10</u>**. For more clarity, we have now rewritten **<u>paragraph 1.4 p.9-10</u>** including the percentages and quantifications in the text (in red).

• The manuscript does not provide a rationale for the choice of fixing the global coupling (G=1). Since G is generally regarded as a crucial parameter that influences global network dynamics, wouldn't it be beneficial to include it in the iterative fitting process alongside local parameters? It would be helpful if the authors provided a justification for why G is held constant.

<u>Answer:</u> The rationale for the choice of fixing the value of the global coupling parameter to 1 is that the fitting of this value is contained inside the GEC fitting step, indeed:

$$\frac{dx}{dt} = (a - x_i^2 - y_i^2)x_i - \omega_i y_i + G\sum_{j=1}^{d} C_{ij}(x_j - x_i) + \sigma\eta_i(t)$$

is the same as

$$\frac{dx}{dt} = (a - x_i^2 - y_i^2)x_i - \omega_i y_i + \sum_{j=1}^{d} J_{ij}(x_j - x_i) + \sigma\eta_i(t)$$

where $J_{ij} = G \times C_{ij}$

We fit the product directly $G \times C_{ij}$ instead of splitting it. We have now clarified that in the manuscript in **section 3.7.1 p.16**, for the Hopf model:

"We chose to keep the global coupling parameter set to G=1 because we fitted the entire connectivity matrix C (section 3.8); thus, any changes in the global coupling intensity are integrated in the fitting of the connectivity matrix. From a mathematical point of view, G being a constant, this is the same as rewriting equation (1) as:

$$\frac{dx}{dt} = (a - x_i^2 - y_i^2)x_i - \omega_i y_i + \sum_{j=1}^{d} J_{ij}(x_j - x_i) + \sigma\eta_i(t)$$

$$\frac{dy}{dt} = (a - x_i^2 - y_i^2)y_i - \omega_i x + \sum_{j=1}^{d} J_{ij}(y_j - y_i) + \sigma\eta_i(t)$$

with $J_{ij} = G \times C_{ij}$ "

And in **section 3.7.2 p.17** for the AHP model:

"Similarly as for the Hopf model, we directly fit a connectivity matrix J_{ij} = G x C_{ij} integrating the global coupling into the fitted connectivity matrix."

• The Methods section would benefit from greater transparency and detail. Key metrics, such as the structural similarity index (SSIM), are referenced but not explicitly defined. Including a brief description of the SSIM formula would make the study self-contained.

Answer: We have now added in the **methods section 3.8, p.18**, the definition of the SSIM:

"The SSIM index is a measure of similarity between two images (or matrices) of the same size [Wang et al. 2004]. It is computed by comparing common-size windows of corresponding zones of both matrices and then aggregating the results. The formula used for the comparison of two matrices A and B is:

$$SSIM(A, B) = \frac{(2\mu_A\mu_B + c_1)(2\sigma_{AB} + c_2)}{(\mu_A^2+\mu_B^2+c_1)(\sigma_A^2+\sigma_B^2+c_2)}$$

where:

μ_X is the pixel empirical mean of matrix X in {A,B} and σ_X its empirical variance;

σ_{AB} the covariance of the two matrices;

c_1 and c_2 are two variables introduced to stabilize the division with a weak denominator, we used the classical default values:

$c_1 = (0.01L)^2$ and $c_2 = (0.03L)^2$ where L is the dynamic range of pixel values in A and B, which in our case is L=1.0".

Similarly, the procedures for fitting model parameters and the rationale behind the specific GEC formula are not described with sufficient clarity. While it is reasonable to reference prior work for context, the paper would be easier to follow if these core methods were explicitly presented.

Answer: We have now extended the paragraph regarding the GEC in the **methods section 3.8, p.19** to make it more self-contained:

*“**Generative Effective Connectivity fitting** Briefly, the generative effective connectivity matrix (GEC) is a matrix that allows for the reproduction of both the empirical correlation matrix of the data and a forward time-shifted correlation matrix. This is done to grasp the asymmetries in the effective connectivity between the nodes and leads to an asymmetric matrix which can reproduce non-equilibrium dynamics (see for example [Sanz Perl et al. 2023, Idesis et. al 2024] for more details about GEC matrices). In practice, we fitted the GEC by keeping the node parameters from the previous step* Θ *fixed and by adjusting the connectivity matrix to maximize the SSIM between the empirical* FC^{emp} *vs simulated* FC^{sim} *matrices, as well as between the shifted matrices* $FC^{emp\text{-}tau}$ *and* $FC^{sim\text{-}tau}$*. The shifted matrices were obtained by shifting in time each of the time series of a small time step tau and taking its correlation with the unshifted other dimensions. The GEC was fitted iteratively using a pseudo gradient descent algorithm according to equation (6) below, which weighs both the fitting of the direct and shifted FC matrices, as also explained in [Sanz Perl et al. 2023]:”*

• Beyond methodological issues, the manuscript’s writing could be improved to enhance readability. The use of inconsistent tenses, punctuation errors, and occasional typos (e.g., “… simulations is Nmax is reached”, or “. Where the shifted matrices are obtained..”, or “using the edgeboundary function”) detract from the clarity of the scientific content. I recommend using tools like Grammarly to identify and correct these issues. Additionally, some sentences are overly verbose or unclear, such as “Moreover, analysis of the clusters’ separability also revealed which parameter, or group of parameters, were leading to belong to which cluster” which could be rephrased more concisely. A thorough revision for grammar, style (e.g., reducing the excessive use of the Saxon genitive 's), and logical flow would make the paper more accessible to the reader and better communicate its scientific value.

Answer: We have now corrected these errors and rewritten the unclear sentences.

• A revised title might help improve the paper's visibility. The current title, “Modeling disorders of consciousness at the patient level reveals the network's influence on the diagnosis vs the local node parameters role in prognosis”, feels a bit lengthy. Additionally, using “vs” is not ideal. You might consider something like: “Personalized models of Disorders of Consciousness reveal complementary roles of connectivity and local parameters in diagnosis and prognosis”.

Answer: This is a very good suggestion, thank you. We have now changed the title as proposed!

• The figures, while generally informative, also require some adjustments. Missing labels, such as for the clusters’ IDs, or the absent color bar in functional connectivity figures, should be added to ensure clarity. In Figure 4A, the yellow cluster is nearly invisible, making it difficult to interpret the results. Adjusting the color palette or improving the visual contrast would address this issue. Figure S2 title

should be “GEC-based biomarkers for the Hopf model:” and panel C should be adjusted in the caption “bf C.”.

Answer: We have now made the suggested corrections. The revised figures are **Figures 3 & 5** in the main text, and **S2, S3 & S4 in the SI**. Plus, there is **now an additional figure, Fig. 4**.

- Notice that PONE guidelines specify that “If there are restrictions on publicly sharing data […] those must be specified.”

Answer: We will share the data necessary to replicate our results (while complying with the protective measures enforced by the clinical IRBs).

In conclusion, this study presents a promising framework for analyzing DoC patients, combining innovative methodologies with biologically grounded insights. However, substantial revisions are required to address the issues outlined above. Expanding the connectivity analysis to both datasets or justifying their combined analysis would enhance the manuscript's robustness, while clearer interpretations and improved methodology transparency will help realize the full potential of this promising framework. I hope that these comments will assist in improving the manuscript.
Sincerely,
Giovanni Rabuffo

Thank you for your very relevant comments and suggestions. They really improved the manuscript. We hope that in the present form it is now suited for publication in Plos ONE.

Best regards,
Lou Zonca, on behalf of all the co-authors.
