# Supplementary material for: Personalized models of disorders of consciousness reveal complementary roles of connectivity and local parameters in diagnosis and prognosis
Source: PLoS One. 2025 Sep 2;20(9):e0328219. doi: 10.1371/journal.pone.0328219 (PMC12404413; doi:10.1371/journal.pone.0328219)
Supplement: Attachment — Submitted filename: PONE-D-24-54958_reviewer_GR_2_response.pdf [file pone.0328219.s005.pdf]

Second Review PONE-D-24-54958

I would like to thank the authors for their thorough responses to all my previous comments. The revised manuscript reflects the substantial effort made to address the points I raised. I appreciate the clarifications incorporated throughout the text and the additions, in particular the analysis connecting GEC cliques and latent dimensions to res<ng-state networks. This strengthens the interpretability of the model.

Answer: Thank you, these were very relevant comments and we really do feel that this has improved our manuscript's quality !

That said, I still have a few minor suggestions for revision before final acceptance.

Minor Revisions

- **Clarification on the use of $G = 1$ in the model**: Thank you for the clarification — this was my misunderstanding. While I initially requested this, the inclusion of the reformulated equations (Eq 2) may be unnecessary.. a brief men<on might have sufficed.

Answer: Agreed, we have removed (Eq 2) and left a short explanation for the choice of G=1.

- **Language polishing**: The manuscript still contains a few minor typos and grammatical errors. These do not detract from the scientific content but should be corrected for clarity.
  (Examples: "empirical func3onal FC .." —> "empirical FC …", "two-dimensionality reduc3on methods..." —> "two dimensionality-reduc3on methods…", "each of these categories include.." —> "each of these categories includes.." , etc.)
  Also, please check for inconsistent figure references (e.g., "fig X" vs. "Fig X" vs. "Figure X").
  I recommend running the manuscript through a proofreading tool such as Grammarly for a final pass.

Answer: We have now corrected the typos and errors, and verified the consistency of figure references.

- **Figure resolution**: Several figures in the PDF appear pixelated or low-resolution. Please ensure that all figures are submitted in high resolution for publication.

Answer: Thank you for highlighting this, we have added a vectorial version of all figures to ensure best quality.

- **Missing Figure 0**: I was unable to view Figure 0, which is referenced in the introduction and abstract as a graphical summary. Please ensure it is included in the final version or clarify how it can be accessed.

Answer: Sorry to hear about that, we have now verified that the graphical abstract is available both in tif and pdf, like all the other figures.

In conclusion, this is a strong and well-executed study. The modeling approach is original and carefully implemented, and the authors have now provided sufficient clarification on all major points raised. With the minor corrections listed above, I am happy to recommend this paper for publication.

Thank you for your kind remarks !

Sincerely,
Giovanni Rabuffo
